# A novel statistical framework for quantifying risks and benefits of AI automation in screening mammography

**DOI:** 10.1371/journal.pdig.0001231

**Published:** 2026-02-26

**Authors:** Michael H. Bernstein, Maggie Chung, Adam Yala, Grayson L. Baird

**Affiliations:** 1 Brown Radiology Human Factors Lab, Department of Radiology, The Warren Alpert Medical School, Brown University, and Brown University Health, Providence, Rhode Island, United States of America; 2 Department of Radiology and Biomedical Imaging, University of California, San Francisco, California, United States of America; 3 Program in Computational Precision Health, University of California, Berkeley and University of California, San Francisco, California, United States of America; Durham University, UNITED KINGDOM OF GREAT BRITAIN AND NORTHERN IRELAND

## Abstract

AI has been proposed as a triage or “rule-out” device to reduce radiologist workload, but it is presently unclear how an AI “rule-out” threshold should be determined. We present a framework for determining an optimal threshold. Using a retrospective study design, 114,229 bilateral 2D digital screening mammograms were analyzed from 2006-2023 at a single study site. All mammograms were given an AI score using Mirai, an open-source deep-learning model which provides a 1-year risk score. Several metrics were examined using two thresholds for determining ruled out versus retained cases: 1) Caseload Reduction Rate (CRR; percent of caseload reduced due to rule-out), 2) Gross AI False Omission Rate (G-FOR; probability of a patient having breast cancer if ruled out), 3) AI Net False Omission Rate (N-FOR; probability of a patient having breast cancer if ruled out and the radiologist would have caught in standard care [i.e., no triage]), 4) AI Adjusted Net False Omission Rate (30%) (AN-FOR[30%]; N-FOR adjusted for the hypothetical scenario where radiologists detect an extra 30% of breast cancers among AI retained cases). The two thresholds were risk scores of 0.2 (Youden’s J) and 0.05 (AN-FOR[30%]=0). The former is mathematically optimal; the latter reflects a threshold where AI “rule-out” does not introduce any total increase in False Negatives. At the 0.20 threshold, G-FOR, N-FOR, and AN-FOR (30%) are 0.26%, 0.17%, and 0.14%, respectively (223, 141, and 121, respectively, missed cancer cases) and CRR = 75%. At the 0.05 threshold, the G-FOR, N-FOR, and AN-FOR (30%) are 0.12%, 0.07%, and 0.00% (49, 30, and 0, respectively, missed cancer cases) and CRR = 36%. We demonstrate how radiology practices can consider the trade-offs of using different AI scores as “rule-out” thresholds. At the AN-FOR rate of 30%, the Youden’s J threshold results in 121 additional missed cancers for a 75% caseload reduction. We estimate no additional missed cancers at a 36% caseload reduction.

## Author summary

Radiology practices are facing rapidly rising imaging volume and workforce shortages. One proposed approach is to use AI to “rule out” the lowest-risk screening mammograms to allow radiologists to focus on a smaller set of exams that are more likely to contain cancer. However, it is unclear how rule-out thresholds should be selected (i.e., how to determine which proportion of cases should be ruled out versus retained for interpretation). We present a statistical framework to quantify the trade-offs between workload reduction and diagnostic risk when considering different potential thresholds for AI rule-out in triage. Using risk scores from 114,229 screening mammograms and an open-source AI model (Mirai), we translate different AI score thresholds into error rates that are clinically relevant and interpretable, and also calculate the workload reduction rate at each threshold. Importantly, our framework distinguishes additional cancers that AI “rule-out” would miss, from cancers that would have also been missed under standard practice without AI “rule-out”. We also consider how using AI “rule-out” may impact radiologist performance such as the possibility that radiologists may detect more cancers among retained cases when the reading pool is smaller and enriched. Our framework is an illustration of how different threshold choices can produce different balances of caseload reduction and net missed cancers. Rather than recommending a specific threshold, this work provides a generalizable tool for practices and policymakers to evaluate AI “rule-out” strategies using local data and specific risk tolerances.

## Introduction

The increasing use of artificial intelligence (AI) in radiology has prompted considerations about its potential in addressing the field’s mounting challenges. In recent years, the workload of radiologists has grown significantly. For instance, one study found that the workload for on-call radiologists in the Emergency Department quadrupled between 2006 and 2020 [1]. Another study that examined billed work relative value units (RVUs) among more than 35,000 academic radiologists found a 60% increase in workload from 2008 to 2020 [2]. This growing burden contributes to rising rates of burnout [3,4]. Furthermore, the number of individuals entering radiology residency has not kept pace with the rise of imaging volume [5], creating a growing workforce imbalance that is unlikely to be resolved in the near future.

AI has the potential to alleviate some of this burden. In particular, one promising application to improve efficiency is using AI as a triage or “ rule-out” device. By identifying screening mammograms that are extremely low-risk, AI can reduce the number of cases that require interpretation by a radiologist. The overwhelming majority of screening mammogram studies (>99%) are cancer-negative given the low prevalence of breast cancer in the screening population. As a result, radiologists devote the vast majority of their screening interpretation time to normal exams. Reductions in the number of low-risk cases requiring radiologist interpretation could translate into large cumulative time savings and workload reduction. This “rule-out” approach is well-suited for pathologies with a low prevalence rate, where there are many true negative cases that can be ruled out at the cost of very few false negatives. Thus, AI rule-out may be suited for screening mammograms where fewer than 1% of cases are positives [6,7]. The goal is to safely exclude the majority of normal cases and allow radiologists to concentrate on more suspicious exams.

Several groups have proposed using AI triage for “rule-out” in radiology; in breast imaging, studies have suggested that AI “rule-out” can perform comparably to, and in some cases better than, standard of care where radiologists interpret all mammograms [8–15]. It also aligns with a growing recognition that successful deployment of AI in medical imaging should not simply assist radiologists within the same interpretive task, but should instead take on distinct, complementary responsibilities that are aligned with each party’s strengths [16]. AI risk and detection models are increasingly evaluated in prospective, workflow-integrated settings that extend beyond interpretive assistance, including early implementation work exploring their use in prioritizing screening mammography workflows [17,18]. The potential role of AI rule-out of negative exams is an active area of discussion among regulatory bodies and professional societies. In this evolving landscape, a rigorous quantitative framework for evaluating potential AI “rule-out” thresholds, such as the one proposed in this study, may help inform future policy and implementation efforts.

One critical component of AI “rule-out” is determining the appropriate **Triage Threshold.** Most AI algorithms generate a continuous risk score for each image, with higher scores indicating a greater likelihood of pathology. However, where the precise cut-off should be placed for distinguishing which cases are ruled out (i.e., non-triage) versus reviewed by a radiologist (i.e., triage) remains an open question [19–22]. Setting the threshold depends on balancing a variety of benefits and risks, which we discuss below. For clarity, these are divided into “ruled out” and “retained” cases. Prior studies have typically selected thresholds based on conventional diagnostic performance metrics (e.g., sensitivity, specificity, ROC-AUC) or workload-reduction targets [8–12], but do not qualify the error burden introduced by a proposed rule-out policy and its downstream workflow impact compared to standard of care.

In this article, we present a framework for determining the optimal AI “rule-out” threshold for screening mammogram automation. We outline key metrics to evaluate the trade-offs between benefits and risks at different thresholds. Specifically, using AI risk scores from the Mirai model applied to 114,229 screening mammograms, we simulate rule-out thresholds to quantify their effects on caseload reduction and cancer detection. We propose approaches for identifying the optimal threshold based on these metrics. Importantly, the objective of this study is not to assess the performance of a specific AI system or threshold, nor to recommend a particular operating point. Rather, the goal of this study is to present a general framework for estimating false-positive and false-negative error rates in the context of AI “rule-out” as a function of caseload reduction relative to standard practice (i.e., no AI).

## Methods

### Ethics statement

University of California, San Francisco (UCSF) Institutional Review Board gave ethical approval for this Health Insurance Portability and Accountability Act–compliant study and waived the requirement for written informed consent.

### Operational definitions

#### Overview.

Below, and in Table 1, we provide an operational definition for all terms used throughout the manuscript. A key consideration of this analysis is that examining metrics of AI performance, in isolation, is insufficient, because it fails to account for important counterfactuals and the AI-radiologist interaction (e.g., did AI fail to detect a pathology that the radiologist would have detected under standard care, or would the radiologist have also missed it?)

**Table 1:** Key Metrics and Definitions.

Metric	Definition
**Caseload Reduction Rate (CRR)**	The percentage of screening mammograms that would have been read by a radiologist under standard care but were excluded from review due to AI-based “rule-out”.
**AI Negative Predictive Value (AI-NPV)**	Probability of a patient not having breast cancer if ruled out by AI.
**AI Positive Predictive Value (AI-PPV)**	Probability of a patient having breast cancer, given that the case was retained for radiologist review.
**AI False Discovery Rate (AI-FDR)**	Probability of a patient not having breast cancer, given that the case was retained for radiologist review.
**AI Gross False Omission Rate** **(G-FOR)**	Probability of a patient having breast cancer if ruled out by AI.
**AI Net False Omission Rate** **(N-FOR)**	Probability of a patient having breast cancer if ruled out by AI, excluding cases that would have also been missed by radiologists without “rule-out”.
**AI Adjusted Net False Omission Rate (AN-FOR)**	Probability of a patient having breast cancer if ruled out, excluding cases that would have also been missed by radiologists, adjusted for breast cancer cases that AI retained but radiologists would have missed without “rule-out”.

#### Benefits and risks in ruled-out cases.

The primary benefit of ruling out cases is caseload reduction, which can be quantified as the **Caseload Reduction Rate (CRR) (**Table 1). The higher the threshold, the higher the CRR; that is, the caseload reduction for radiologists will be higher when a more stringent (i.e., higher) threshold is set, ruling out a larger pool of cases. However, the benefit of a higher CRR must be weighed against a variety of other considerations.

First, one must consider how accurate an AI is at correctly ruling out cancer, given the cancer prevalence in the population; this is reflected by the **AI Negative Predictive Value (AI-NPV)**. AI-NPV is the probability that a patient ruled out by AI truly does not have breast cancer. Some negative cases ruled out by AI might have otherwise been recalled by the radiologist in standard care (interpreting mammograms without AI triaging), potentially leading to unnecessary, costly, and stress-inducing diagnostic imaging and biopsies that turn out to be benign.

The aforementioned benefit must be carefully weighed against the **AI Gross False Omission Rate (G-FOR, or 1-AI-NPV),** which is the probability that a patient ruled out by AI actually has breast cancer. As the threshold is raised to exclude more cases, the G-FOR increases. That is, the CRR and the G-FOR come at a clear tradeoff; the more cases AI rules out, the higher the G-FOR will be. Nonetheless, it is important to note that not all cancers missed by AI in the ruled-out cases would have been detected by radiologists under **standard practice (SP**) (i.e., radiologist workflow absent an AI triaging model). That is, some cancer cases would likely have been missed regardless of whether AI triaging was used. To account for this, we define the **AI Net False Omission Rate (N-FOR)** as the G-FOR minus cancer cases that would have been missed by AI and radiologists (i.e., “deduct” cancer cases mutually missed by both radiologists and AI “rule-out” from the numerator).

#### Benefits and risks in retained cases.

The **AI Positive Predictive Value (AI-PPV)** reflects the probability that a patient has breast cancer given that the case was retained for radiologist review. The **AI False Discovery Rate (AI-FDR, or 1-AI-PPV)** refers to the probability that a patient retained by AI for radiologist review does not actually have breast cancer. The higher the “rule-out” threshold, the more cases AI will rule-out (i.e., the larger the CRR). This means that remaining (i.e., retained) cases are more likely to be true positives, which increases the AI-PPV and reduces the AI-FDR. However, decreasing the number of retained cases (and by definition also increasing the number of ruled-out cases) can have important implications for how they are interpreted.

Radiologist performance may improve as the retained reading pool size decreases due to reading fewer cases [23], reading an enriched batch with higher prevalence [24–26], and by consciously or unconsciously knowing that the cases were triaged [22] (i.e., anchoring or automation bias). These additional cancer detections could potentially offset a portion of the cancers missed among ruled-out cases due to the use of AI “rule-out” (N-FOR). Taking this into account, the **Adjusted Net False Omission Rate (AN-FOR)** refers to the probability of a patient having breast cancer that would have been detected by a radiologist in standard practice if ruled out, adjusted for the additional cancer detections due to AI “rule-out” that would have been missed in standard practice (i.e., “credit” cancer cases that radiologists would have otherwise missed without AI triaging them).

Another risk worth considering is that although radiologists are more likely to catch cancer cases they would have otherwise missed had AI not retained them, it is also likely that for the same reason, radiologists may also increase unnecessary recalls (i.e., radiologists recall non-cancer cases they would not have otherwise recalled had they not been retained by triage) [22].

### Simulation methods

To illustrate the trade-offs associated with different AI “rule-out” thresholds, we conducted a “simulation” using risk scores from a deep learning model with screening mammography.

Study Sample. We conducted a single institution retrospective review of 114,229 bilateral 2D digital screening mammograms acquired between January 2006 and January 2023. Only screening examinations with at least 12 months of imaging follow-up within our health system were included; exams without complete follow-up were excluded from all analyses. All examinations in the cohort included standard 2D digital mammography images; a subset of exams also included tomosynthesis (DBT). Radiologists interpreted exams using the full available clinical dataset, including DBT when available. Exams with histopathologically confirmed breast cancer within 12 months of the screening mammogram were considered positive. Exams with at least 12 months of follow-up without a breast cancer diagnosis were considered negative. Based on these criteria, 864 cases (0.76%) were identified as positive. Of the positive cancers, screen-detected cancers were defined as examinations in which the patient was recalled at the index screening mammogram and subsequently diagnosed with breast cancer. Cancers were classified as “missed by radiology” (interval cancers) if the patient was not recalled at the index screening exam but was diagnosed with breast cancer within 12 months.

AI Model. Mammograms were assessed using Mirai, an open-source deep learning model available at https://github.com/yala/Mirai. All analyses were performed using the publicly released v0.5.0 codebase. No local modifications to the model architecture or weights were made [27,28]. Mirai was trained exclusively on non-UCSF data, with complete institutional separation from the evaluation cohort and no risk of data leakage. One-year risk scores (henceforth “scores” or “Mirai scores”) were used to simulate “rule-out” thresholds. The Mirai model accepts 2D digital mammography images only, and therefore only 2D images were used as inputs for all simulations.

Threshold Simulation Framework. We simulated various “rule-out” thresholds based on Mirai scores.

Modeling Assumptions. To model AN-FOR, we simulated four possible scenarios in which 10%, 30%, 50%, or 70% of missed cancers in standard practice were detected by using AI “rule-out”. These are intended as hypothetical scenarios only; the true rate is unknown. Likewise, our results utilize an AN-FOR rate of 30%, but this is for illustrative purposes only and should not be interpreted as suggesting 30% is the value that best corresponds with clinical practice.

Statistics. All modeling was conducted using SAS 9.4 (SAS Cary, NC), where sensitivities and specificities were estimated using the LOGISTIC procedure with the %ROCPLOT macro, and PPV, NPV, FDR, and FOR were calculated using Bayes’ Theorem (see Appendix A for PPV and NPV equations). The base rate of cancer was 0.76%. Confidence limits were generated for FDR and FOR using bootstrapping with the SURVEYSELECT procedure using 1000 replicates.

## Results

### Approaches to identifying rule-out threshold

Data were simulated using two “rule-out” thresholds that can be generalized across practices. The first uses diagnostic performance—Youden’s J—to define a threshold by optimizing the balance of sensitivity and specificity. The second defines a threshold using an outcome, in this case, avoiding any overall increase in missed breast cancers compared to standard practice without triage. That is, this threshold is set so that an AN-FOR of 0 is achieved, meaning all cancers missed by using AI triage (rule-out cases) are then offset by an identical number of cancer cases that a radiologist would catch because they were retained.

### Identifying threshold using diagnostic performance (Youden’s J)

For these data, we observed that the Youden’s J value is a Mirai score of 0.20, achieving a sensitivity of 74% and a specificity of 75% (see Tables 2 and 4). Given a local prevalence of 0.76%, this translated into ruling out 85,220 cases and retaining 29,009 cases, resulting in a CRR of 75% (85,220/114,229). Of these ruled-out cases, 223 had breast cancer and 84,997 did not, thus achieving a G-FOR of 223/85,220 (0.26%). Of the retained cases, 641 had breast cancer and 28,368 did not, thus achieving an AI-FDR of 97.8%.

**Table 2:** Error Rates Using Youden’s J Threshold.

All Cases					
Youden’s J		Breast Cancer			Error Rates
Threshold	AI Decision	No	Yes	Total	
**≤.20**	**Rule-out**	84,997	223	85,220	G-FOR: 223/85,220
**>.20**	**Retain**	28,368	641	29,009	AI-FDR: 28,368/29,009
	Total	113,365	864	114,229	
**AI Rule-out Cases**					
**≤.20**	**Radiologist Recall**	**No**	**Yes**	**Total**	
	No	79,194	82	79,276	
	Yes	5,803	**141**	5,944	Numerator for N-FOR: 141
		84,997	223	85,220	Denominator for all FORs: 85,220
**AI Retained Cases**					
**>.20**	**Radiologist Recall**	**No**	**Yes**	**Total**	
	No	24,995	**66**	25,061	Numerator for AN-FOR (30%): 121
	Yes	3,373	575	3,948	
		28,368	641	29,009	

Note: 141- (66 X.30) = 121.

**Table 3:** Key for Table 4.

Threshold		Breast Cancer			Error Rates
	Decision	No	Yes	Total	
**≤.A**	**Rule-out**	F-(C + F)	C + E	F	G-FOR: (C + E)/F
**>.A**	**Retain**	K-(H + K)	H + J	K	AI-FDR: [K-(H + K)]/K
**Rule-out**	**Decision**	**No**	**Yes**	**Total**	
*Radiologist* *Recall*	No	B	C	B + C	
	Yes	D	E	D + E	Numerator for G-FOR: E
**Retain**	**Decision**	**No**	**Yes**	**Total**	
*Radiologist* *Recall*	No	G	H	G + H	Numerator for N-FOR: (E-H)
	Yes	I	J	I + J	

**Table 4:** Table of metrics and outcomes.

A	Rule-out					Retained						GFOR	NFOR	AN-FOR %				FDR	CRR
Score	B	C	D	E	F	G	H	I	J	K	Total	%	%	10%	30%	50%	70%	%	%
0.01						104189	148	9176	716	114229	114229							99.24	
0.02	10691	1	593	2	11287	93498	147	8583	714	102942	114229	0.03	0.02	-0.11	-0.37	-0.63	-0.89	99.16	9.88
0.03	22684	6	1345	11	24046	81505	142	7831	705	90183	114229	0.07	0.05	-0.01	-0.13	-0.25	-0.37	99.06	21.05
0.04	31603	13	1932	18	33566	72586	135	7244	698	80663	114229	0.09	0.05	0.01	-0.07	-0.15	-0.23	98.97	29.38
**0.05**	**38634**	**19**	**2444**	**30**	**41127**	**65555**	**129**	**6732**	**686**	**73102**	**114229**	**0.12**	**0.07**	**0.04**	**-0.02**	**-0.08**	**-0.15**	**98.89**	**36.00**
0.06	44185	27	2882	40	47134	60004	121	6294	676	67095	114229	0.14	0.08	0.06	0.01	-0.04	-0.09	98.81	41.26
0.07	48879	36	3239	46	52200	55310	112	5937	670	62029	114229	0.16	0.09	0.07	0.02	-0.02	-0.06	98.74	45.70
0.08	52957	42	3565	56	56620	51232	106	5611	660	57609	114229	0.17	0.10	0.08	0.04	0.01	-0.03	98.67	49.57
0.09	56547	44	3837	65	60493	47642	104	5339	651	53736	114229	0.18	0.11	0.09	0.06	0.02	-0.01	98.59	52.96
0.10	59695	48	4109	75	63927	44494	100	5067	641	50302	114229	0.19	0.12	0.10	0.07	0.04	0.01	98.53	55.96
0.11	62487	51	4337	84	66959	41702	97	4839	632	47270	114229	0.20	0.13	0.11	0.08	0.05	0.02	98.46	58.62
0.12	65099	57	4541	93	69790	39090	91	4635	623	44439	114229	0.21	0.13	0.12	0.09	0.07	0.04	98.39	61.10
0.13	67432	62	4741	98	72333	36757	86	4435	618	41896	114229	0.22	0.14	0.12	0.10	0.08	0.05	98.32	63.32
0.14	69548	65	4906	105	74624	34641	83	4270	611	39605	114229	0.23	0.14	0.13	0.11	0.09	0.06	98.25	65.33
0.15	71511	71	5081	113	76776	32678	77	4095	603	37453	114229	0.24	0.15	0.14	0.12	0.10	0.08	98.18	67.21
0.16	73310	75	5246	117	78748	30879	73	3930	599	35481	114229	0.24	0.15	0.14	0.12	0.10	0.08	98.11	68.94
0.17	74938	77	5387	125	80527	29251	71	3789	591	33702	114229	0.25	0.16	0.15	0.13	0.11	0.09	98.04	70.50
0.18	76506	80	5530	130	82246	27683	68	3646	586	31983	114229	0.26	0.16	0.15	0.13	0.12	0.10	97.96	72.00
0.19	77918	80	5657	135	83790	26271	68	3519	581	30439	114229	0.26	0.16	0.15	0.14	0.12	0.10	97.87	73.35
**0.20**	**79194**	**82**	**5803**	**141**	**85220**	**24995**	**66**	**3373**	**575**	**29009**	**114229**	**0.26**	**0.17**	**0.16**	**0.14**	**0.13**	**0.11**	**97.79**	**74.60**
0.21	80400	85	5909	147	86541	23789	63	3267	569	27688	114229	0.27	0.17	0.16	0.15	0.13	0.12	97.72	75.76
0.22	81607	93	6021	154	87875	22582	55	3155	562	26354	114229	0.28	0.18	0.17	0.16	0.14	0.13	97.66	76.93
0.23	82692	94	6130	161	89077	21497	54	3046	555	25152	114229	0.29	0.18	0.17	0.16	0.15	0.14	97.58	77.98
0.24	83731	100	6250	167	90248	20458	48	2926	549	23981	114229	0.30	0.19	0.18	0.17	0.16	0.15	97.51	79.01
0.25	84690	102	6352	175	91319	19499	46	2824	541	22910	114229	0.30	0.19	0.19	0.18	0.17	0.16	97.44	79.94
0.26	85677	104	6446	179	92406	18512	44	2730	537	21823	114229	0.31	0.19	0.19	0.18	0.17	0.16	97.34	80.90
0.27	86525	106	6545	186	93362	17664	42	2631	530	20867	114229	0.31	0.20	0.19	0.19	0.18	0.17	97.26	81.73
0.28	87354	109	6639	189	94291	16835	39	2537	527	19938	114229	0.32	0.20	0.20	0.19	0.18	0.17	97.16	82.55
0.29	88174	109	6742	198	95223	16015	39	2434	518	19006	114229	0.32	0.21	0.20	0.20	0.19	0.18	97.07	83.36
0.30	88942	110	6822	205	96079	15247	38	2354	511	18150	114229	0.33	0.21	0.21	0.20	0.19	0.19	96.98	84.11
0.31	89608	111	6904	212	96835	14581	37	2272	504	17394	114229	0.33	0.22	0.22	0.21	0.20	0.19	96.89	84.77
0.32	90256	113	6992	217	97578	13933	35	2184	499	16651	114229	0.34	0.22	0.22	0.21	0.20	0.20	96.79	85.42
0.33	90865	113	7069	225	98272	13324	35	2107	491	15957	114229	0.34	0.23	0.23	0.22	0.21	0.20	96.70	86.03
0.34	91440	115	7146	231	98932	12749	33	2030	485	15297	114229	0.35	0.23	0.23	0.22	0.22	0.21	96.61	86.61
0.35	92040	116	7203	236	99595	12149	32	1973	480	14634	114229	0.35	0.24	0.23	0.23	0.22	0.21	96.50	87.19
0.36	92621	117	7280	243	100261	11568	31	1896	473	13968	114229	0.36	0.24	0.24	0.23	0.23	0.22	96.39	87.77
0.37	93160	118	7342	248	100868	11029	30	1834	468	13361	114229	0.36	0.25	0.24	0.24	0.23	0.23	96.27	88.30
0.38	93678	119	7404	255	101456	10511	29	1772	461	12773	114229	0.37	0.25	0.25	0.24	0.24	0.23	96.16	88.82
0.39	94170	120	7462	259	102011	10019	28	1714	457	12218	114229	0.37	0.25	0.25	0.25	0.24	0.23	96.03	89.30
0.40	94655	123	7529	269	102576	9534	25	1647	447	11653	114229	0.38	0.26	0.26	0.25	0.25	0.25	95.95	89.80
0.41	95197	123	7593	275	103188	8992	25	1583	441	11041	114229	0.39	0.27	0.26	0.26	0.25	0.25	95.78	90.33
0.42	95665	124	7667	279	103735	8524	24	1509	437	10494	114229	0.39	0.27	0.27	0.26	0.26	0.25	95.61	90.81
0.43	96148	125	7725	284	104282	8041	23	1451	432	9947	114229	0.39	0.27	0.27	0.27	0.26	0.26	95.43	91.29
0.44	96594	126	7791	294	104805	7595	22	1385	422	9424	114229	0.40	0.28	0.28	0.27	0.27	0.27	95.29	91.75
0.45	96997	127	7872	299	105295	7192	21	1304	417	8934	114229	0.40	0.28	0.28	0.28	0.27	0.27	95.10	92.18
0.46	97421	129	7926	310	105786	6768	19	1250	406	8443	114229	0.41	0.29	0.29	0.29	0.28	0.28	94.97	92.61
0.47	97800	129	7987	317	106233	6389	19	1189	399	7996	114229	0.42	0.30	0.30	0.29	0.29	0.29	94.77	93.00
0.48	98195	130	8032	323	106680	5994	18	1144	393	7549	114229	0.42	0.30	0.30	0.30	0.29	0.29	94.56	93.39
0.49	98554	130	8075	333	107092	5635	18	1101	383	7137	114229	0.43	0.31	0.31	0.31	0.30	0.30	94.38	93.75
0.50	98903	131	8128	344	107506	5286	17	1048	372	6723	114229	0.44	0.32	0.32	0.32	0.31	0.31	94.21	94.11
0.51	99244	132	8182	353	107911	4945	16	994	363	6318	114229	0.45	0.33	0.33	0.32	0.32	0.32	94.00	94.47
0.52	99574	135	8223	362	108294	4615	13	953	354	5935	114229	0.46	0.33	0.33	0.33	0.33	0.33	93.82	94.80
0.53	99874	136	8285	367	108662	4315	12	891	349	5567	114229	0.46	0.34	0.34	0.33	0.33	0.33	93.52	95.13
0.54	100186	138	8338	374	109036	4003	10	838	342	5193	114229	0.47	0.34	0.34	0.34	0.34	0.34	93.22	95.45
0.55	100440	138	8381	375	109334	3749	10	795	341	4895	114229	0.47	0.34	0.34	0.34	0.34	0.34	92.83	95.71
0.56	100726	138	8429	389	109682	3463	10	747	327	4547	114229	0.48	0.35	0.35	0.35	0.35	0.35	92.59	96.02
0.57	100980	138	8479	397	109994	3209	10	697	319	4235	114229	0.49	0.36	0.36	0.36	0.36	0.35	92.23	96.29
0.58	101278	138	8520	405	110341	2911	10	656	311	3888	114229	0.49	0.37	0.37	0.36	0.36	0.36	91.74	96.60
0.59	101504	138	8555	412	110609	2685	10	621	304	3620	114229	0.50	0.37	0.37	0.37	0.37	0.37	91.33	96.83
0.60	101721	138	8596	419	110874	2468	10	580	297	3355	114229	0.50	0.38	0.38	0.38	0.37	0.37	90.85	97.06
0.61	101936	138	8637	428	111139	2253	10	539	288	3090	114229	0.51	0.39	0.38	0.38	0.38	0.38	90.36	97.29
0.62	102142	138	8669	439	111388	2047	10	507	277	2841	114229	0.52	0.39	0.39	0.39	0.39	0.39	89.90	97.51
0.63	102334	139	8700	450	111623	1855	9	476	266	2606	114229	0.53	0.40	0.40	0.40	0.40	0.40	89.45	97.72
0.64	102525	139	8734	463	111861	1664	9	442	253	2368	114229	0.54	0.41	0.41	0.41	0.41	0.41	88.94	97.93
0.65	102681	139	8767	472	112059	1508	9	409	244	2170	114229	0.55	0.42	0.42	0.42	0.42	0.42	88.34	98.10
0.66	102847	140	8799	480	112266	1342	8	377	236	1963	114229	0.55	0.43	0.43	0.43	0.42	0.42	87.57	98.28
0.67	103014	141	8833	489	112477	1175	7	343	227	1752	114229	0.56	0.43	0.43	0.43	0.43	0.43	86.64	98.47
0.68	103153	142	8864	496	112655	1036	6	312	220	1574	114229	0.57	0.44	0.44	0.44	0.44	0.44	85.64	98.62
0.69	103276	143	8906	504	112829	913	5	270	212	1400	114229	0.57	0.45	0.45	0.45	0.44	0.44	84.50	98.77
0.70	103384	143	8941	512	112980	805	5	235	204	1249	114229	0.58	0.45	0.45	0.45	0.45	0.45	83.27	98.91
0.71	103491	145	8975	523	113134	698	3	201	193	1095	114229	0.59	0.46	0.46	0.46	0.46	0.46	82.10	99.04
0.72	103608	145	8996	535	113284	581	3	180	181	945	114229	0.60	0.47	0.47	0.47	0.47	0.47	80.53	99.17
0.73	103694	145	9018	541	113398	495	3	158	175	831	114229	0.60	0.48	0.48	0.48	0.48	0.48	78.58	99.27
0.74	103763	145	9043	553	113504	426	3	133	163	725	114229	0.61	0.49	0.49	0.49	0.49	0.49	77.10	99.37
0.75	103817	145	9053	565	113580	372	3	123	151	649	114229	0.63	0.50	0.50	0.50	0.50	0.50	76.27	99.43
0.76	103871	145	9068	580	113664	318	3	108	136	565	114229	0.64	0.51	0.51	0.51	0.51	0.51	75.40	99.51
0.77	103918	145	9082	591	113736	271	3	94	125	493	114229	0.65	0.52	0.52	0.52	0.52	0.52	74.04	99.57
0.78	103954	145	9095	602	113796	235	3	81	114	433	114229	0.66	0.53	0.53	0.53	0.53	0.53	72.98	99.62
0.79	104003	145	9108	612	113868	186	3	68	104	361	114229	0.66	0.54	0.54	0.54	0.54	0.54	70.36	99.68
0.80	104025	145	9117	622	113909	164	3	59	94	320	114229	0.67	0.55	0.55	0.55	0.54	0.54	69.69	99.72
0.81	104063	146	9128	628	113965	126	2	48	88	264	114229	0.68	0.55	0.55	0.55	0.55	0.55	65.91	99.77
0.82	104081	146	9135	633	113995	108	2	41	83	234	114229	0.68	0.56	0.56	0.55	0.55	0.55	63.68	99.80
0.83	104098	146	9141	641	114026	91	2	35	75	203	114229	0.69	0.56	0.56	0.56	0.56	0.56	62.07	99.82
0.84	104114	147	9148	653	114062	75	1	28	63	167	114229	0.70	0.57	0.57	0.57	0.57	0.57	61.68	99.85
0.85	104132	147	9156	657	114092	57	1	20	59	137	114229	0.70	0.58	0.58	0.58	0.58	0.58	56.20	99.88

Of the 223 breast cancer cases that were ruled out, 82 were not recalled. That is, 82 were also missed by radiologists in standard of care while they recalled the remaining 141, thus achieving an N-FOR of 141/85,220 or 0.17%.

**Fig 1:**
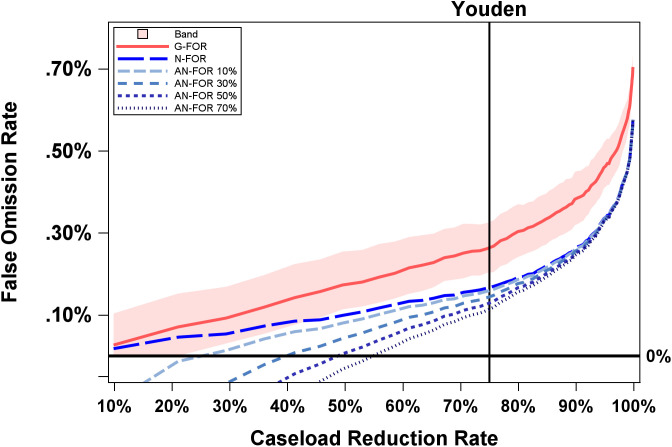
False Omission Rate by Caseload Reduction Rate. X-axis is caseload reduction rate (10% to 100%) and Y-axis is False Omission Rate (0.0% to 0.70%). Youden refers to Youden’s J (thin black line). G-FOR is Gross False Omission Rate (solid red). N-FOR is Net False Omission Rate (longest dash, bright blue). AN-FOR 10% (long dash, light blue), AN-FOR 30% (short dash, medium blue), AN-FOR 50% (short dash, dark blue), AN-FOR 70% (shortest dash, grey blue) refer to the Adjusted Net False Omission Rate at various percentages of additional breast cancers that radiologists would detect (10%, 30%, 50%, and 70% respectively) in AI-retained cases using an AI “rule-out” model relative to standard of care. Bootstrapped 95% confidence interval curve is shown in red-shaded area.

Regarding the retained cases, AI retained 66 cases that radiologists missed. Assuming radiologists detect 10%, 30%, 50% or 70% of these cases in AI “rule-out”, the adjusted net number of missed cancers in AI “rule-out” would be reduced to 7, 20, 33, or 46–134, 121, 108, or 95, respectively. This would correspond to Adjusted Net FOR values of 0.16%, 0.14%, 0.13%, and 0.11%, respectively. These values are visualized in Table 4 and Fig 1 (with invasive-cancer–only results shown in S1 Fig).

### Identifying threshold using outcomes

Another approach to identifying the threshold is by considering the type of error and number of errors that would result from AI triage based on historical data. As shown in Fig 1 and Tables 3 and 4, depending on the percentage of additional breast cancer cases (i.e., 10%, 30%, 50%, and 70%) that radiologists would have detected among those retained by AI “rule-out” (compared to standard of care), the rule-out threshold can be set by determining the caseload reduction rate where AN-FOR intersects a certain value (here 0). As discussed above, this threshold corresponds to no additional missed cancers overall (among both retained and ruled out cases) relative to standard practice. As illustrated in Fig 1 and Table 4 (**bold**), assuming radiologists detect an additional 30% of missed cancers in cases retained by AI, a threshold of Mirai = 0.05 would achieve an AN-FOR of 0, which would translate into a CRR of about 36%. If radiologists detect an additional 70% of missed cancers, a threshold of Mirai = 0.09 would achieve an AN-FOR of 0, which would translate into a CRR of about 53%.

**Fig 2:**
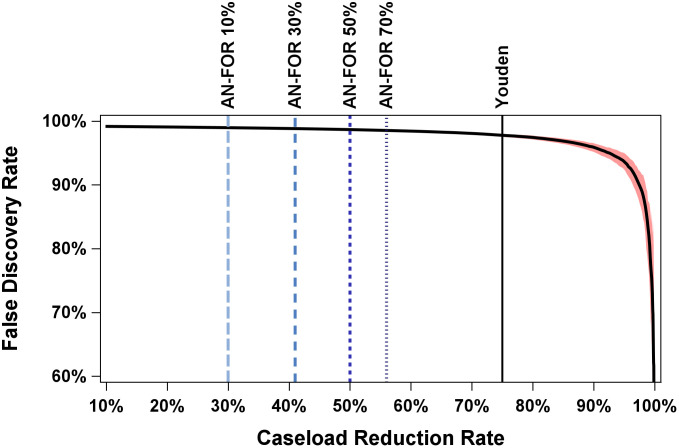
False Discovery Rate by Caseload Reduction Rate. X-axis is caseload reduction rate (10% to 100%) and Y-axis is False Discovery Rate (60% to 100%). Thick black line is the relationship between Caseload Reduction Rate and False Discovery Rate Youden refers to Youden’s J (thin black line). AN-FOR 10% (long dash, light blue), AN-FOR 30% (short dash, medium blue), AN-FOR 50% (short dash, dark blue), AN-FOR 70% (shortest dash, grey blue) refer to the Adjusted Net False Omission Rate at various percentages of additional breast cancers that radiologists would detect (10%, 30%, 50%, and 70% respectively) in AI-retained cases using an AI “rule-out” model relative to standard of care. Bootstrapped 95% confidence interval curve is shown in red-shaded area.

These CRR values can then be used to examine the corresponding number of false positives. As seen in Fig 2, the AI-FDR was between about 98% and 99% for all thresholds considered, indicating that FDR was largely stable. Given that false positives are unlikely to vary significantly, mainly because of low cancer prevalence [21], false negatives will be the primary focus here. Invasive-cancer–only results are shown in S2 Fig. To illustrate downstream clinical impact, we also quantified the number of benign and high-risk biopsies that would be avoided under each AI rule-out threshold. As shown in S1 Table, increasing caseload reduction is associated with a corresponding increase in potentially avoidable biopsies among ruled-out cases.

### Comparing thresholds

To assess the trade-off between errors and benefits, we compare two thresholds: Mirai score of 0.20 corresponding to a 75% CRR (Youden’s J) and Mirai score of 0.05 corresponding to a 36% caseload reduction assuming AN-FOR of 0 where 30% of additional breast cancers would have been detected among retained cases by radiologists using an AI “rule-out” model compared to standard of care.

At the 0.20 threshold, the G-FOR, N-FOR, and AN-FOR (30%) are 0.26%, 0.17%, and 0.14%, respectively. This corresponds to 223, 141, and 121 missed cancer cases for the benefit of reading 85,220 fewer cases with an FDR of 97.8%. In contrast, at the 0.05 threshold, the G-FOR, N-FOR, and AN-FOR (30%) are 0.12%, 0.07%, and 0.00% (rounded), corresponding to 49, 30, and 0 missed cancer cases for the benefit of reading 41,127 fewer cases with an FDR of 98.9%. Tables 3 and 4 provide all combinations for comparison.

## Discussion

We demonstrate how radiology practices can consider the trade-offs of using different AI scores to determine the “rule-out” threshold. Using the Mirai AI algorithm and historical data, our simulation demonstrates how a risk-benefit analysis could be quantified. Crucially, the purpose of this framework is not to advocate for a specific threshold or risk-benefit ratio or to evaluate a particular AI system. Rather, the goal of the present study is to demonstrate how a risk-benefit ratio could be quantified to inform policy and clinical implementation of AI “rule-out”. All numerical values provided are illustrative and are not intended as recommendations for clinical use.

The optimal threshold will vary depending on the AI model, the pathology (and the corresponding trade-offs of false positives and false negatives), the AI model’s sensitivity and specificity for a local population, the prevalence of the local population, the local caseload volume and radiologist staffing ability, and institutional risk tolerances. Our simulation highlights how error rates (risk) and caseload reduction rate (benefit) can be estimated using historical data. This estimation not only accounts for the **type of errors (i.e., false positive and false negative)** but also the **number of errors (i.e., false discovery and omission rates instead of false positive and negative rates)**.

Our proposed framework could also be generalized to these screening domains. Many accepted screening programs operate in populations with disease prevalence below 1%. For example, low-dose CT lung cancer screening detects malignancy in 0.4-0.9% of examinations [29,30]. Abdominal ultrasound screening for abdominal aortic aneurysm finds clinically significant aneurysms requiring intervention in less than 0.5% of screened patients [31]. Thus, across modalities, the overwhelming majority (>99%) of screening examinations are disease-negative or findings that do not require intervention.

Research examining the performance of AI in radiology typically rely on sensitivity, specificity, and AUC-ROC metrics [8–12]. Error rates are critical for interpreting AI feedback. Fan et al. propose evaluating AI “rule-out” using PPV and NPV. The current study builds upon their approach in two key ways. Namely, Fan et al. do not account for key counterfactuals such as cancers that would have been missed by radiologists without triage and cancer only detected with triage because of changes in radiologist performance [13]. As such, the degree to which AI “rule-out” impacts diagnostic performance relative to no AI “rule-out” is not fully captured. In addition, Fan et al. propose using expected utility (EU) to assess AI “rule-out”. However, this relies on baseline relative utility values, which are difficult to define and when defined, may be difficult to justify, economically, ethically, and otherwise.

While our simulation focused on the number of any missed cancers, the type (e.g., in situ versus invasive) and stages/grade of cancers missed by AI could be incorporated to further assess the clinical significance of triage-related errors. What is more, we only evaluated cancers diagnosed within a year of the screening mammogram; other time frames (e.g., 1 and 2-year cancer outcomes) could be incorporated as well. Finally, for simplicity, we calculated the G-FOR, N-FOR, AN-FOR, and FDR using the direct rates, although confidence, prediction, or credible interval estimates could be used instead. Establishing specific thresholds or noninferiority margins is beyond the scope of this study as it would require multi-stakeholder policy decisions incorporating institutional risk tolerance, legal and regulatory context, radiologist availability, patient preferences, and operational priorities. However, our framework provides a quantitative approach for exploring and tailoring these decisions to individual practice settings. Additional limitations include: the use of only one study site, hypothetical percents used for AN-FOR values, the lack of a prospective study validation, and the lack of subanalyses by density or age. Moreover, the downstream effects of FDR (e.g., biopsy rate) may not be constant across different “rule-out” thresholds, a consideration that is not captured in this study. In addition, some patients may have undergone cancer diagnosis beyond the 12-month follow-up window and therefore were not captured in our outcome definition. Importantly, these limitations primarily affect the precision and generalizability of the illustrative numerical results and do not alter the central contribution of this work, which is the presentation of a general conceptual framework for evaluating AI “rule-out” strategies.

## Future directions and implications

Along with a framework for determining a threshold for AI “rule-out” of screening mammograms, there are several important considerations that must be addressed before AI “rule-out” can be implemented in clinical practice. First, prospective validation of AI rule-out strategies is needed. In this study, we evaluated a range of hypothetical values (10–70%, with a focal scenario of 30%), which, while illustrative, highlight a critical unknown that must be resolved empirically. Past research has shown that automation bias plays a critical role in radiology [22], and other work has demonstrated that increased prevalence results in more abnormal images being flagged [25]. The AN-FOR rate will likely change as a function of threshold, and future work is needed to more carefully consider that level of nuance. Examining this topic empirically would require a multi-case multi-reader (MCMR) design where radiologists interpret imaging without AI, and then with AI at different “rule-out” thresholds. This validation will be important for understanding how AI “rule-out” impacts radiologist performance in the retained cases. Also, when local data are examined historically, practices should be mindful of how changes in technology, prevalence rate, and workflow may impact performance metrics and select the appropriate retrospective window. From a regulatory perspective (e.g., Food and Drug Administration or European Medicines Agency), one option might be to set the “rule-out” threshold at the point where AN-FOR rate is 0, after first running the algorithm on local data and estimating the relevant AN-FOR percentage, using the aforementioned MCMR study design. This would translate to caseload reduction without a net increase in the number of FNs. By quantifying error rates attributable to rule-out and accounting for downstream effects in retained cases, this framework may assist regulatory bodies in more comprehensively assessing benefit–risk profiles, informing study design, operating-point selection, and post-market monitoring strategies for AI triage “rule-out” systems.

Second, standards need to be developed for the safe deployment of AI “rule-out” tools in clinical settings and address approaches for ongoing monitoring of AI performance and safety over time. Third, there are psychological, ethical, legal, economic, and insurance considerations that must be weighed if implementing “rule-out”. Finally, there will need to be significant changes to the policy and regulatory landscape to allow AI “rule-out” in clinical practice. Addressing these considerations is necessary for the implementation of AI “rule-out”.

## Supporting information

S1 Fig False Omission Rate by Caseload Reduction Rate For Invasive Cancers. False omission rate (FOR) plotted against caseload reduction rate when restricting outcomes to invasive cancers only. The x-axis denotes caseload reduction rate (10–100%), and the y-axis denotes false omission rate (0–0.70%). The thin black vertical line indicates the threshold selected by Youden’s J statistic. The solid red curve represents the gross false omission rate (G-FOR), and the solid bright-blue curve represents the net false omission rate (N-FOR). Dashed curves show the adjusted net false omission rate (AN-FOR), assuming that radiologists detect an additional 10%, 30%, 50%, or 70% of cancers in AI-retained cases relative to standard of care (light blue long dash, medium blue short dash, dark blue short dash, and gray-blue shortest dash, respectively).
(DOCX)

S2 Fig False Discovery Rate as a Function of Caseload Reduction for Invasive Cancers. False discovery rate (FDR) plotted against caseload reduction rate when restricting outcomes to invasive cancers only. The x-axis denotes caseload reduction rate (10–100%), and the y-axis denotes false discovery rate (60–100%). The thick black curve represents the empirical relationship between caseload reduction and FDR. The thin black vertical line indicates the threshold selected by Youden’s J statistic. Dashed curves represent adjusted net false omission rate (AN-FOR) scenarios assuming that radiologists detect an additional 10%, 30%, 50%, or 70% of cancers in AI-retained cases relative to standard of care (light blue long dash, medium blue short dash, dark blue short dash, and gray-blue shortest dash, respectively).
(DOCX)

S1 Table Biopsies Avoided at Each AI Rule-Out Threshold. Number of biopsies resulting in high-risk or benign pathology that would be retained or ruled out at each AI score threshold. For each threshold, counts are reported separately for high-risk and benign pathology. Values in the “ruled-out” columns represent biopsies that could be avoided under the corresponding AI rule-out strategy, whereas values in the “retained” columns represent biopsies that would remain in the clinical workflow and be performed downstream of a screening recall.
(DOCX)
