# Supplementary figures and images for: A novel statistical framework for quantifying risks and benefits of AI automation in screening mammography
Source: PLOS Digit Health. 2026 Feb 26;5(2):e0001231. doi: 10.1371/journal.pdig.0001231 (PMC12944777; doi:10.1371/journal.pdig.0001231)

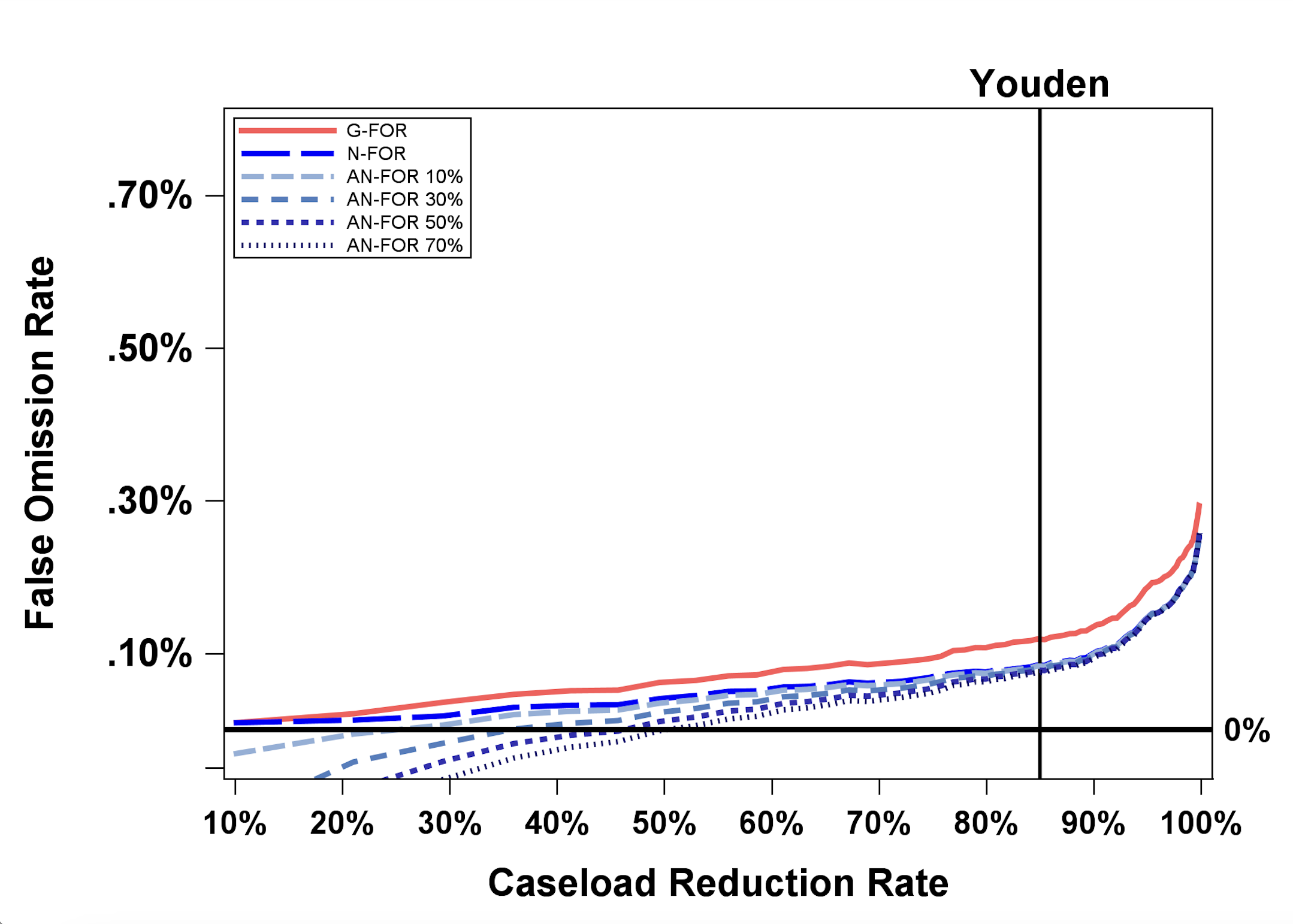


S1 Fig

Supplement: S1 Fig — False omission rate (FOR) plotted against caseload reduction rate when restricting outcomes to invasive cancers only. The x-axis denotes caseload reduction rate (10–100%), and the y-axis denotes false omission rate (0–0.70%). The thin black vertical line indicates the threshold selected by Youden’s J statistic. The solid red curve represents the gross false omission rate (G-FOR), and the solid bright-blue curve represents the net false omission rate (N-FOR). Dashed curves show the adjusted net false omission rate (AN-FOR), assuming that radiologists detect an additional 10%, 30%, 50%, or 70% of cancers in AI-retained cases relative to standard of care (light blue long dash, medium blue short dash, dark blue short dash, and gray-blue shortest dash, respectively). (DOCX) [file pdig.0001231.s001.docx]

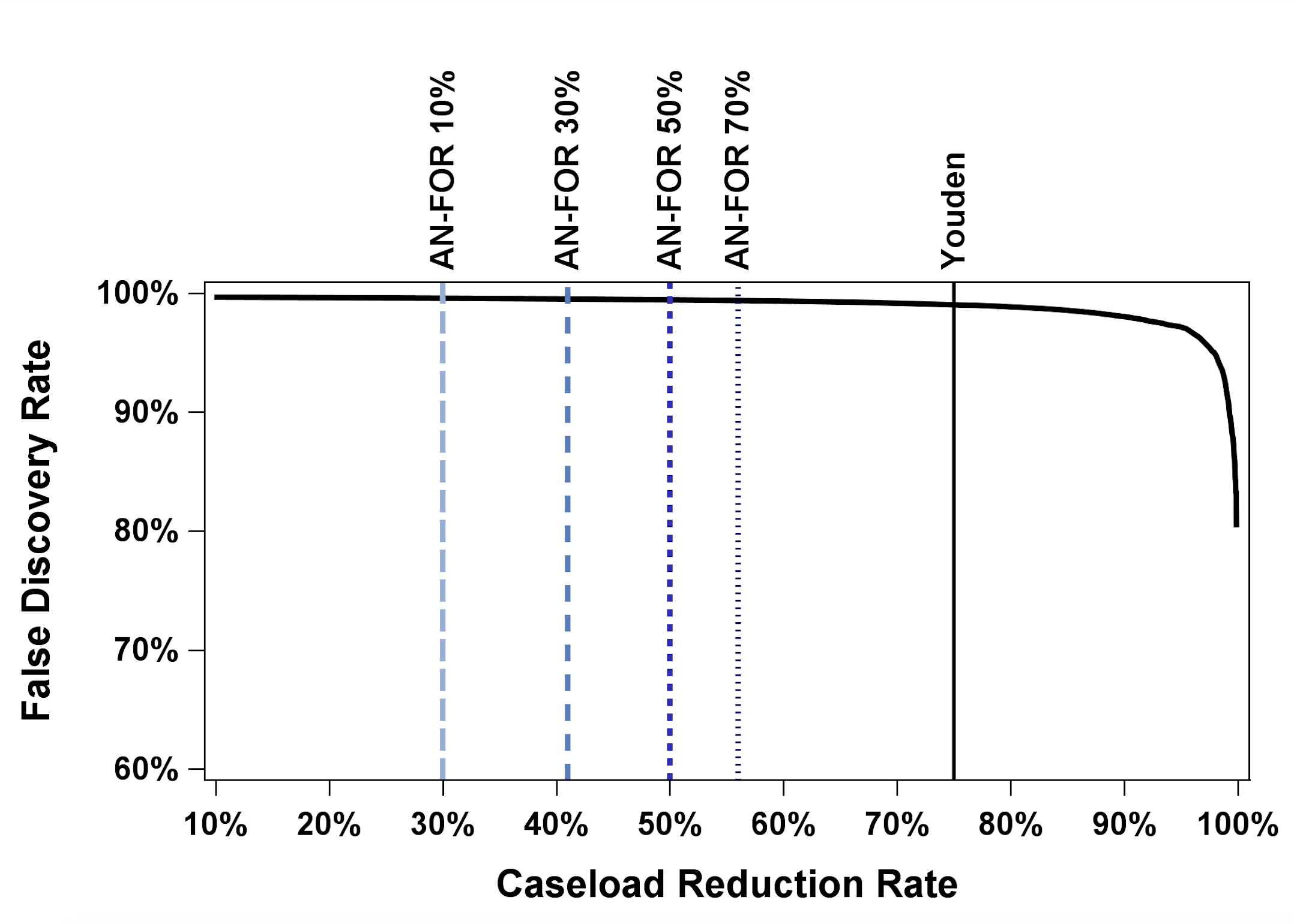


S2 Fig

Supplement: S2 Fig — False discovery rate (FDR) plotted against caseload reduction rate when restricting outcomes to invasive cancers only. The x-axis denotes caseload reduction rate (10–100%), and the y-axis denotes false discovery rate (60–100%). The thick black curve represents the empirical relationship between caseload reduction and FDR. The thin black vertical line indicates the threshold selected by Youden’s J statistic. Dashed curves represent adjusted net false omission rate (AN-FOR) scenarios assuming that radiologists detect an additional 10%, 30%, 50%, or 70% of cancers in AI-retained cases relative to standard of care (light blue long dash, medium blue short dash, dark blue short dash, and gray-blue shortest dash, respectively). (DOCX) [file pdig.0001231.s002.docx]
