# Supplementary material for: A novel statistical framework for quantifying risks and benefits of AI automation in screening mammography
Source: PLOS Digit Health. 2026 Feb 26;5(2):e0001231. doi: 10.1371/journal.pdig.0001231 (PMC12944777; doi:10.1371/journal.pdig.0001231)
Supplement: S1 Table — Number of biopsies resulting in high-risk or benign pathology that would be retained or ruled out at each AI score threshold. For each threshold, counts are reported separately for high-risk and benign pathology. Values in the “ruled-out” columns represent biopsies that could be avoided under the corresponding AI rule-out strategy, whereas values in the “retained” columns represent biopsies that would remain in the clinical workflow and be performed downstream of a screening recall. (DOCX) [file pdig.0001231.s003.docx]

S1 Table

|  | High Risk Biopsies | | Benign Biopsies | |
| --- | --- | --- | --- | --- |
| AI score threshold | Retained | Ruled-out | Retained | Ruled-out |
| 0.01 | 301 |  | 1651 |  |
| 0.02 | 300 | 1 | 1589 | 62 |
| 0.03 | 293 | 8 | 1488 | 163 |
| 0.04 | 288 | 13 | 1399 | 252 |
| 0.05 | 283 | 18 | 1323 | 328 |
| 0.06 | 274 | 27 | 1258 | 393 |
| 0.07 | 266 | 35 | 1214 | 437 |
| 0.08 | 262 | 39 | 1160 | 491 |
| 0.09 | 259 | 42 | 1122 | 529 |
| 0.1 | 251 | 50 | 1082 | 569 |
| 0.11 | 248 | 53 | 1058 | 593 |
| 0.12 | 244 | 57 | 1028 | 623 |
| 0.13 | 241 | 60 | 998 | 653 |
| 0.14 | 236 | 65 | 977 | 674 |
| 0.15 | 231 | 70 | 944 | 707 |
| 0.16 | 228 | 73 | 926 | 725 |
| 0.17 | 223 | 78 | 901 | 750 |
| 0.18 | 217 | 84 | 875 | 776 |
| 0.19 | 211 | 90 | 850 | 801 |
| 0.2 | 207 | 94 | 822 | 829 |
| 0.21 | 203 | 98 | 802 | 849 |
| 0.22 | 199 | 102 | 777 | 874 |
| 0.23 | 194 | 107 | 753 | 898 |
| 0.24 | 186 | 115 | 729 | 922 |
| 0.25 | 186 | 115 | 703 | 948 |
| 0.26 | 184 | 117 | 688 | 963 |
| 0.27 | 176 | 125 | 666 | 985 |
| 0.28 | 172 | 129 | 644 | 1007 |
| 0.29 | 170 | 131 | 618 | 1033 |
| 0.3 | 166 | 135 | 605 | 1046 |
| 0.31 | 161 | 140 | 588 | 1063 |
| 0.32 | 156 | 145 | 573 | 1078 |
| 0.33 | 152 | 149 | 559 | 1092 |
| 0.34 | 150 | 151 | 537 | 1114 |
| 0.35 | 146 | 155 | 526 | 1125 |
| 0.36 | 143 | 158 | 509 | 1142 |
| 0.37 | 138 | 163 | 499 | 1152 |
| 0.38 | 135 | 166 | 489 | 1162 |
| 0.39 | 131 | 170 | 472 | 1179 |
| 0.4 | 125 | 176 | 453 | 1198 |
| 0.41 | 124 | 177 | 441 | 1210 |
| 0.42 | 121 | 180 | 424 | 1227 |
| 0.43 | 120 | 181 | 409 | 1242 |
| 0.44 | 120 | 181 | 397 | 1254 |
| 0.45 | 117 | 184 | 375 | 1276 |
| 0.46 | 111 | 190 | 366 | 1285 |
| 0.47 | 107 | 194 | 349 | 1302 |
| 0.48 | 101 | 200 | 338 | 1313 |
| 0.49 | 97 | 204 | 331 | 1320 |
| 0.5 | 95 | 206 | 318 | 1333 |
| 0.51 | 93 | 208 | 300 | 1351 |
| 0.52 | 90 | 211 | 284 | 1367 |
| 0.53 | 81 | 220 | 271 | 1380 |
| 0.54 | 78 | 223 | 262 | 1389 |
| 0.55 | 75 | 226 | 255 | 1396 |
| 0.56 | 73 | 228 | 242 | 1409 |
| 0.57 | 70 | 231 | 231 | 1420 |
| 0.58 | 67 | 234 | 220 | 1431 |
| 0.59 | 65 | 236 | 207 | 1444 |
| 0.6 | 64 | 237 | 190 | 1461 |
| 0.61 | 62 | 239 | 179 | 1472 |
| 0.62 | 60 | 241 | 172 | 1479 |
| 0.63 | 57 | 244 | 162 | 1489 |
| 0.64 | 53 | 248 | 152 | 1499 |
| 0.65 | 51 | 250 | 146 | 1505 |
| 0.66 | 47 | 254 | 138 | 1513 |
| 0.67 | 44 | 257 | 129 | 1522 |
| 0.68 | 41 | 260 | 120 | 1531 |
| 0.69 | 35 | 266 | 108 | 1543 |
| 0.7 | 31 | 270 | 94 | 1557 |
| 0.71 | 29 | 272 | 82 | 1569 |
| 0.72 | 28 | 273 | 74 | 1577 |
| 0.73 | 23 | 278 | 67 | 1584 |
| 0.74 | 19 | 282 | 59 | 1592 |
| 0.75 | 18 | 283 | 54 | 1597 |
| 0.76 | 17 | 284 | 48 | 1603 |
| 0.77 | 17 | 284 | 40 | 1611 |
| 0.78 | 16 | 285 | 36 | 1615 |
| 0.79 | 13 | 288 | 30 | 1621 |
| 0.8 | 10 | 291 | 29 | 1622 |
| 0.81 | 9 | 292 | 23 | 1628 |
| 0.82 | 9 | 292 | 19 | 1632 |
| 0.83 | 9 | 292 | 17 | 1634 |
| 0.84 | 8 | 293 | 13 | 1638 |
| 0.85 | 6 | 295 | 10 | 1641 |
